# High-purity quantum optomechanics at room temperature

**DOI:** 10.1038/s41567-025-02976-9

**Published:** 2025-08-06

**Authors:** Lorenzo Dania, Oscar Schmitt Kremer, Johannes Piotrowski, Davide Candoli, Jayadev Vijayan, Oriol Romero-Isart, Carlos Gonzalez-Ballestero, Lukas Novotny, Martin Frimmer

**Affiliations:** 1 Photonics Laboratory, ETH Zürich, Zurich, Switzerland; 2 Quantum Center, ETH Zürich, Zurich, Switzerland; 3 ICFO - Institut de Ciencies Fotoniques, The Barcelona Institute of Science and Technology, Barcelona, Spain; 4 Photon Science Institute, Department of Electrical and Electronic Engineering, University of Manchester, Manchester, UK; 5 ICREA - Institucio Catalana de Recerca i Estudis Avancats, Barcelona, Spain; 6 Institute for Theoretical Physics and Vienna Center for Quantum Science and Technology, Vienna University of Technology (TU Wien), Vienna, Austria

**Keywords:** Quantum mechanics, Optical manipulation and tweezers, Quantum metrology, Nanosensors

## Abstract

Exploiting quantum effects in a mechanical oscillator, such as back-action-evading measurements or squeezing of the mechanical degrees of freedom, requires the oscillator to be prepared in a high-purity quantum state. The largest state purities in optomechanics to date have been achieved with costly cryogenic cooling combined with coupling to electromagnetic resonators driven with a coherent radiation field. Here we use coherent scattering into a Fabry–Pérot cavity to cool the megahertz-frequency librational mode of an optically levitated silica nanoparticle from room temperature to its quantum ground state. We use sideband thermometry to infer a phonon population of 0.04 quanta under optimal conditions, corresponding to a state purity of 92%. The purity reached by our room-temperature experiment exceeds the performance offered by mechanically clamped oscillators in a cryogenic environment, establishing a platform for high-purity quantum optomechanics at room temperature.

## Abstract (web summary)

Observing quantum effects in a mechanical oscillator requires it to be close to a pure quantum state, rather than a thermal mixture. Here a librational mode of a levitated nanoparticle is cooled close to its ground state without using cryogenics.

## Main

The prospect of observing and exploiting quantum states of massive systems has been driving the field of optomechanics^1^. Mechanical motion controlled by optical or microwave fields offers opportunities to develop quantum-enhanced sensing schemes^2–4^ and transduction technologies^5^, test quantum mechanics at unprecedented mass and length scales^6,7^, and gain insights into the role of the gravitational field in the evolution of quantum states^8,9^. Crucial to these applications is the suppression of thermal noise, which calls for operation in a cryogenic environment. Although highly effective, the cost and technical complexity of cryogenic cooling severely limit further proliferation of optomechanical technologies. The promise of room-temperature quantum optomechanics has, therefore, spurred the development of experimental platforms operating without the need for cryogenic cooling^10^.

The necessity to suppress thermal noise is rooted in the requirement of any quantum protocol to initialize the oscillator in a quantum mechanically pure state^11,12^. For a thermal state with mean occupation number *n*, the purity is defined by ${\mathcal{P}}={(2n+1)}^{-1}$ (ref. ^13^). For sufficiently large mechanical frequencies, such as gigahertz mechanical modes of nanobeams^14^ or bulk acoustic-wave resonators^15^, state purification can be achieved by thermalization to a cryogenic bath sufficiently cold to render the oscillator in its ground state of motion. For megahertz or even lower mechanical frequencies, high-purity state preparation requires a combination of cryogenic cooling with other techniques such as cavity sideband cooling^16–18^ or measurement-based feedback cooling^19,20^. With such combined cooling schemes, megahertz electromechanical oscillators have been prepared with a phonon occupation of 0.07 (88% state purity)^16^ and gigahertz optomechanical systems have been brought to a purity of 85% (ref. ^21^).

The key to circumventing the need for cryogenic cooling is to suppress the coupling of the mechanics to its thermal environment. In this vein, two approaches have been followed. The first is based on complete mechanical decoupling of the oscillator from its environment by optical levitation in vacuo^22^. Cooling the centre-of-mass motion of an optically levitated nanoparticle in room-temperature experiments has been reported using both measurement-based feedback^23^ and laser-sideband cooling^24^, reaching a phonon population of 0.6, corresponding to a state purity of 47% (ref. ^23^). The second approach focuses on careful design of the strain and phononic dispersion of the mechanical tether^25,26^. In a tour de force of mechanical and optical engineering, recent efforts have enabled quantum optomechanics at room temperature with a clamped system, reaching a purity of 34% (ref. ^27^). Despite these efforts, no room-temperature optomechanical platform can, at present, rival the state purities achieved with the aid of cryogenics.

Here we report cooling of a megahertz librational mode of an optically levitated nanoparticle to a phonon population of *n* = 0.04 in a room-temperature experiment, corresponding to a state purity ${\mathcal{P}}=92 \%$. Cooling is realized by coupling the nanoparticle to a high-finesse optical cavity in a coherent-scattering configuration^28^. To reach such high purity, we operate our libration-cavity system deep in the sideband-resolved regime, we actively suppress laser phase noise, and we choose the polarization state of the tweezer to maximize the optomechanical coupling rate. Regarding quantum mechanical purity, our results place levitated oscillators in room-temperature experiments ahead of the most performant opto- and electromechanical systems, even those aided by cryogenics and gigahertz mechanical mode frequencies.

## Experimental set-up

**Fig. 1:**
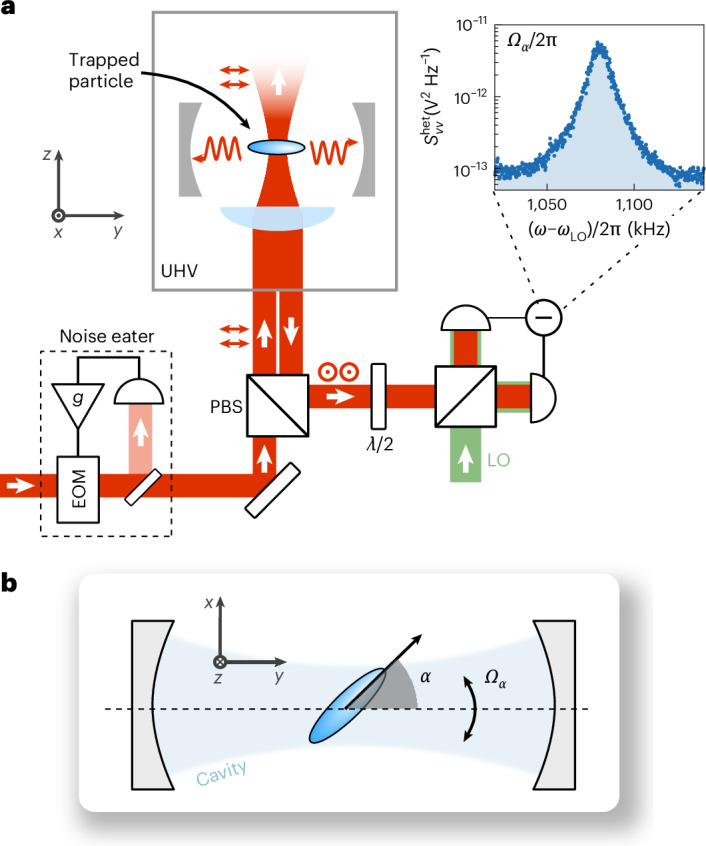
Sketch of the experimental set-up. **a**, An anisotropic silica nanoparticle (schematically illustrated as an ellipsoid) is trapped by an optical tweezer in an ultrahigh vacuum. The tweezer light is linearly polarized along the *y* axis by a polarizing beam splitter. The long axis of the nanoparticle aligns parallel to the tweezer polarization and undergoes angular harmonic oscillations, termed librations, at a frequency *Ω*_*α*_/(2π) in the *x–y* plane. This libration motion is coupled to a high-finesse optical cavity. The high-NA lens forming the optical trap is mounted on a nanopositioner (not shown) such that the particle equilibrium position can be varied across the cavity intensity profile. The *x*-polarized light backscattered from the nanoparticle is collected by the trapping lens and mixed with a local oscillator of frequency *ω*_LO_ in a balanced heterodyne detector. This detector provides a measurement of the libration motion unaltered by the cavity transfer function. Inset: power spectral density ${S}_{VV}^{{\rm{het}}}$ acquired at 6 mbar from this backward detector. The librational mode of the particle peaks at *Ω*_*α*_/(2π) = 1.08 MHz. Laser phase noise in the tweezer beam is suppressed by a noise eater composed of a phase-noise detector and an electro-optic modulator. The suppression level is varied with a gain *g*. **b**, Illustration of the libration mode. The tweezer polarization is aligned to the cavity axis (*y*). The libration angle *α* denotes the deviation of the long axis of the particle from the polarization direction of the tweezer field in the *x–y* plane. EOM, electro-optic modulator; LO, local oscillator; PBS, polarizing beam splitter; UHV, ultrahigh vacuum.

The nanomechanical oscillator investigated in this work is the harmonic angular motion of an optically levitated anisotropic particle^29^. The axis of largest polarizability of an anisotropic scatterer in a linearly polarized field aligns with the polarization direction of the field^30^. Small deviations from this alignment result in harmonic angular motion, termed ‘libration’^31^. Figure 1a shows a sketch of the experimental set-up. More details are given in the Supplementary Information. We use an optical tweezer (power of ~1.2 W, numerical aperture NA = 0.75 and wavelength *λ* = 1,550 nm) to trap single anisotropic nanoparticles inside a vacuum chamber at a pressure of 5 × 10^−9^ mbar and at room temperature. The tweezer beam propagates along the *z* direction and is linearly polarized along the *y* axis, resulting in centre-of-mass frequencies (*Ω*_*x*_, *Ω*_*y*_, *Ω*_*z*_)/(2π) = (250, 220, 80) kHz. We trap anisotropic nanoparticles, which are clusters of a few silica nanospheres. The nanospheres have a nominal diameter of 120 nm. The measured centre-of-mass gas damping rates (Supplementary Information) indicate that each nanoparticle has its long axis aligned to the tweezer polarization^29^ and that its shape is not cylindrically symmetric^32^. We detect the orientation of the nanoparticle by interfering backscattered light from the tweezer with a local oscillator shifted by *ω*_LO_/(2π) = 2.73 MHz in a balanced heterodyne scheme, where *ω*_LO_ is the frequency of the local oscillator. The optical tweezer is positioned in the waist of a high-finesse optical cavity whose axis points along the *y* direction, that is, it is oriented perpendicularly to the tweezer axis and along the polarization direction of the tweezers. Light scattered by the nanoparticle populates the fundamental transverse electromagnetic (TEM_00_) mode of the cavity, resulting in optomechanical coupling through coherent scattering (Supplementary Information). The cavity mode has a linewidth *κ*/(2π) = 330 kHz and a resonance frequency *ω*_c_ = *ω*_tw_ + *Δ*, detuned by *Δ* from the tweezer frequency *ω*_tw_. The nanoparticle position *y*_eq_ along the cavity standing wave is tunable with the nanopositioner holding the trapping lens. Finally, our system contains a noise eater that allows us to controllably suppress laser phase noise^33^.

The anisotropic particle shape results in three distinct moments of inertia (Supplementary Information), giving rise to three non-degenerate libration modes associated with the orientation angles *γ*, *β* and *α*^34,35^, with corresponding frequencies (*Ω*_*γ*_, *Ω*_*β*_, *Ω*_*α*_)/(2π) = (0.15, 0.7, 1.08) MHz. Recent experiments have cooled several libration and centre-of-mass modes simultaneously, either by feedback control^30,32,35^ or by cavity-cooling through coherent scattering^34^. Here we optimize our experimental settings to enhance single-mode cooling of the *α* libration mode.

The high-frequency *α* mode corresponds to angular oscillations in the tweezer focal plane (*x–y* plane in Fig. 1b)^32^. The inset of Fig. 1a shows a high-pressure (6 mbar) spectrum of the heterodyne signal. We associate the Lorentzian peak with the motion of the *α* mode. In this work, we focus on cavity-cooling this mode for two reasons. First, the *α* mode lies deep in the sideband-resolved regime (*Ω*_*α*_ ≫ *κ*), which is a required condition to reach the ground state through cavity-cooling^36^. Second, optimal cooling of the *α* mode is achieved on polarizing the tweezer along the *y* axis (Fig. 1b)^28,37^. Moreover, this configuration directs the dipole radiation pattern of light elastically scattered by a nanoparticle outside the cavity, thus minimizing the heating effects of laser phase noise^38^. As the particle orientation oscillates in the *x–y* plane, light from the tweezer is inelastically scattered into the cavity mode at the Stokes and anti-Stokes frequencies *ω*_tw_ ± *Ω*_*α*_. For a cavity detuning *Δ* ≈ *Ω*_*α*_, the anti-Stokes scattering is enhanced, promoting energy transfer from the mechanics to the light field and resulting in cavity-cooling of the librational motion.

In the following, we detail how we maximize the state purity of our levitated librator. To do this, we first benchmark our thermometry scheme. Then, we optimize the cavity detuning for best cooling performance. Finally, we optimize the particle position in the cavity mode in the presence of phase-noise suppression.

## Sideband thermometry and cavity detuning scan

**Fig. 2:**
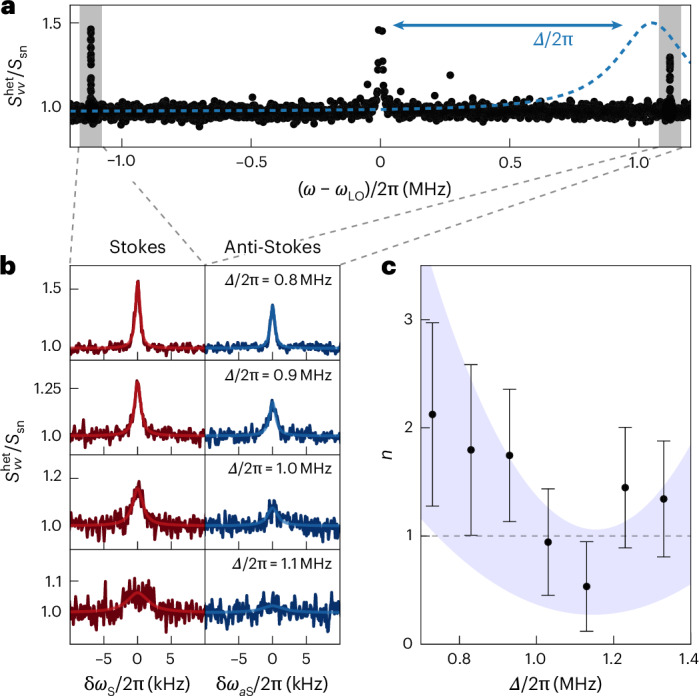
Libration occupation number as a function of cavity detuning. **a**, Heterodyne libration spectrum measured with the free-space backward detector (black), superposed with the cavity lineshape (blue dashed line). The detuning *Δ* is the difference between the tweezer frequency and the cavity resonance frequency. Highlighted in grey are the Stokes and anti-Stokes librational sidebands. For *Δ* ≈ *Ω*_*α*_, with *Ω*_*α*_/(2π) = 1.08 MHz being the librational frequency, the cavity enhances the anti-Stokes scattering, leading to resolved-sideband cooling. **b**, Stokes (left) and anti-Stokes (right) heterodyne spectra ${S}_{VV}^{{\rm{het}}}$ normalized to the shot-noise level *S*_sn_ with fitted Lorentzian lines for *Δ* increasing from top to bottom as indicated in the plot. *δω*_S_ and *δω*_aS_ denote the frequency differences from the Stokes and anti-Stokes peak centres, respectively. **c**, Occupation number *n* obtained from sideband thermometry as a function of *Δ*. It reaches a minimum of *n* = 0.5(3) at *Δ*/(2π) = 1.13 MHz. Occupation values are extracted from Lorentzian fits to the heterodyne spectra. Error bars correspond to one standard deviation of the fitted asymmetries around the calculated occupation numbers. The dashed line marks the *n* = 1 threshold. The shaded area corresponds to a theoretical estimation of *n* based on libration-cavity coupling, laser phase noise, radiation-torque shot noise and their uncertainties (Supplementary Information).

The free-space heterodyne detector gives us access to both Stokes and anti-Stokes sidebands of the *α* librational mode. We confirm cavity-cooling through Raman sideband thermometry^20,23^. Figure 2a shows a heterodyne spectrum of the libration motion (black dots, normalized to the shot-noise level) obtained for cavity detuning *Δ*/(2π) = 0.8 MHz. The plot shows the Stokes (left) and anti-Stokes (right) libration peaks, highlighted by the shaded grey bands. The area *a*_S_ (*a*_aS_) below the Stokes (anti-Stokes) sideband is associated with light-scattering events that increase (decrease) the librational energy. An asymmetry between the two sidebands arises as the angular motion of the particle is cooled close to its ground state. We can deduce the libration occupation number *n* from the ratio of the sidebands *a*_aS_/*a*_S_ = *n*/(*n* + 1) (ref. ^39^). We stress that, unlike schemes detecting the cavity output spectrum^18,24,40^, our free-space detection does not rely on prior knowledge of the cavity detuning and it is insensitive to sideband artefacts from classical laser phase noise, which may corrupt the occupation estimation^41,42^. Furthermore, in the Supplementary Information we rule out the detector transfer function as a possible source of sideband asymmetry.

By using sideband thermometry, we measure the libration occupation number as a function of the cavity detuning. For this measurement, the particle is positioned at *ky*_eq_ ≈ 0.1π in the cavity standing wave, where *k* is the wavenumber, and we define the position along the cavity axis such that *y*_eq_ = 0 coincides with the intracavity intensity minimum. Figure 2b shows heterodyne spectra normalized to the detection shot-noise level *S*_sn_ centred around the Stokes (left column) and anti-Stokes (right column) libration peaks and for increasing values of the cavity detuning *Δ* (top to bottom). To facilitate comparison, the spectra have been shifted along the frequency axis to compensate for the optical spring effect and to align the Stokes and anti-Stokes peaks (Supplementary Information). As the detuning approaches the optimal value *Δ* ≈ *Ω*_*α*_ ≈ 2π × 1.1 MHz, the linewidth increases and the peak height decreases due to cavity-cooling. From a Lorentzian fit (lines in Fig. 2b) to each lineshape, we extract the occupation number *n*.

In Fig. 2c we plot the measured occupation number *n* including the standard deviation of the fits as a function of the cavity detuning *Δ*. We obtain the minimum occupation *n* = 0.5(3) for *Δ* = 2π × 1.1 MHz. To put this result into perspective, we compare *n* to the back-action limit for the phonon population in a coherent-scattering configuration, which is given by ${n}_{{\rm{CS}}}={[\kappa /(4\varOmega )]}^{2}+{n}_{{\rm{BA}}}^{{\rm{fs}}}$. The first term arises due to the interaction of the oscillator with the cavity mode, and the second term ${n}_{{\rm{BA}}}^{{\rm{fs}}}$ is the phonon population due to scattering into free space^43^. An estimate of the back-action limit using our cavity parameters yields ${n}_{{\rm{BA}}}^{{\rm{fs}}}=0.07\gg {[\kappa /(4\varOmega )]}^{2}$, rendering the result in Fig. 2c far from the optimal. To identify our limitation, we show as the blue area in Fig. 2c the theoretical estimate based on our system parameters, including the libration-cavity coupling rate, laser phase noise of the tweezer beam, and radiation-torque shot noise, that is, measurement back-action on a rotor^44^. Our model indicates that the final occupation number in this experiment is limited by laser phase noise in the tweezer beam, a limitation that has plagued previous optomechanics experiments^38,45^. Phase noise is detrimental whenever elastically scattered light at the tweezer frequency populates the cavity, as it turns phase fluctuations into amplitude fluctuations leading to further heating. Elastic scattering into the cavity mode can arise from small experimental imperfections, such as a misalignment between the tweezer polarization and the cavity axis, or a small ellipticity of the tweezer polarization.

## Position dependence of cooling performance

**Fig. 3:**
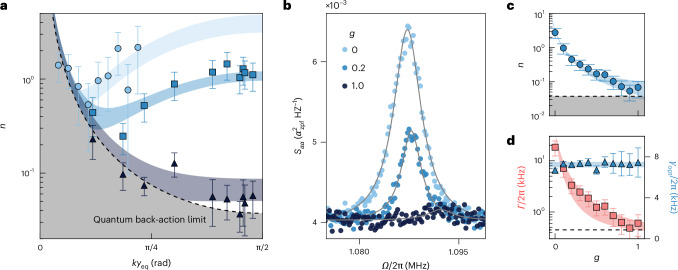
Dependence of cavity-cooling on particle position in the standing wave and phase noise. **a**, Occupation number *n* measured for different particle positions *y*_eq_ along the cavity standing wave and for different levels of phase-noise cancellation *g* as indicated. The cavity intensity minimum coincides with the position *y*_eq_ = 0 (cavity node), and the maximum occurs for *ky*_eq_ = π/2 (cavity anti-node). Occupation values are extracted from Lorentzian fits to calibrated homodyne spectra. The error bars derive from the standard deviation in the Lorentzian fits and from the statistical error on the calibration method. The coloured areas are fits to our model including phase noise, cavity coupling and radiation-torque shot noise (Supplementary Information). The black dashed line corresponds to the quantum back-action limit reachable in the absence of phase noise. **b**, Homodyne spectra *S*_*αα*_ of the libration degree of freedom for different cancellation gains and with the particle at the anti-node. Data are expressed in units of the zero-point angular displacement *α*_zpf_. Grey solid lines are Lorentzian fits. **c**, Occupation number *n* as a function of phase-noise cancellation *g* for a particle in the cavity anti-node at optimal detuning. Occupation values are obtained from a fit to calibrated spectra, and error bars derive from the standard deviation in the fit and statistical errors from the calibration. The blue area represents the theoretical prediction, and the black dashed line is the quantum back-action limit. **d**, Total heating rate *Γ* = *nγ*_opt_ (red) and optical cooling rate *γ*_opt_ (blue) as a function of *g*, extracted from the same dataset underlying **c**. The cooling rates *γ*_opt_ were extracted from Lorentzian fits to the homodyne spectra, and their error bars represent one standard deviation of the fitted parameter. The error bars of *Γ* were obtained by propagating the errors of *n* and *γ*_opt_. The coloured areas following the data points are theoretical predictions based on our model. The black dashed line is the heating rate due to radiation-torque shot noise extracted from the fits in **a**.

To corroborate our understanding, we study the dependence of the cooling performance as a function of particle position in the cavity. Although heating due to laser phase noise is position dependent^24,38^, radiation-torque shot noise and heating from gas collisions^44^ do not depend on position. The blue circles in Fig. 3a show the occupation *n* measured for different positions *y*_eq_ along the standing wave of the cavity field, whose periodicity is set by the wavenumber *k*. This experiment is done at optimal cavity detuning *Δ* ≈ *Ω*_*α*_. We observe that the occupation reaches a minimum value of *n* = 0.5 for *ky*_eq_ ≈ 0.1π. This is the position where the data in Fig. 2c has been acquired. The initially decreasing trend of *n* with position *y*_eq_ is due to the increasing cooling rate when moving away from the intensity minimum of the cavity mode. However, as the particle is placed further away from the node, the cavity photon population builds up and so does heating due to phase noise. This behaviour is quantitatively captured by our model (light-blue area behind the blue circles in Fig. 3a; see Supplementary Information for details), supporting the hypothesis that our cooling performance is limited by phase noise.

## Phase-noise reduction

To combat phase noise in our experiment, we implement a phase-noise eater based on an imbalanced Mach–Zehnder interferometer for noise detection and feedback to the laser through an electro-optic phase modulator^33^. The feedback gain *g* determines the phase-noise suppression, which we characterize in Supplementary Information. The remaining experiments described in this Article are carried out with a different nanoparticle, which has the same libration frequency *Ω*_*α*_ as that used for all experiments presented thus far (Supplementary Information). Furthermore, we implement a quasi-homodyne libration detection scheme^32^ (Supplementary Information) cross-calibrated by sideband thermometry to boost detection efficiency so that we can resolve small occupation numbers.

Figure 3a shows as blue squares the occupation *n* obtained with phase-noise cancellation gain *g* = 0.2. The occupation reaches a minimum value of *n* = 0.25 at a position *ky*_eq_ ≈ 0.2π, closer to the cavity anti-node with respect to the situation without phase-noise cancellation. For gain *g* = 1, shown as black triangles in Fig. 3a, the occupation monotonically decreases and reaches its minimum near the cavity anti-node (*ky*_eq_ ≈ π/2). This is the behaviour expected in the absence of phase noise, where the optimum position of the particle for libration cooling is at the anti-node of the cavity field^28^. The lowest measured occupation is *n* = 0.04(1) quanta, corresponding to a ground-state purity of 92%. The blue and grey areas in Fig. 3a are simultaneous theoretical fits to both data-sets taken with phase-noise cancellation. From these fits, we extract the shot-noise heating rate *Γ*_BA_/(2π) = 0.5(1) kHz. This is the heating rate due to free-space measurement back-action, that is, it arises from scattering of the tweezer beam outside the cavity mode. We use this value to calculate the back-action-limited occupation that can be provided by our system, shown as the dashed black line in Fig. 3a. The proximity of the shot-noise limit to the data taken at cancellation gain *g* = 1 (black triangles) indicates that at this level of laser phase noise (${S}_{\dot{\phi }\dot{\phi }}({\varOmega }_{\alpha })=0.01\,{{\rm{Hz}}}^{2}\,\rm{Hz}^{-1}$; Supplementary Information) the measured occupations are predominantly limited by quantum back-action. Figure 3b is a direct comparison between spectra obtained with and without the noise eater. For these measurements, the particle was positioned at the anti-node, with the same feedback gains used in Fig. 3a. The spectra were calibrated with sideband thermometry and are expressed in units of *α*_zpf_, the zero-point angular displacement (Supplementary Information). We observe how the area below the libration peak shrinks as the gain increases, whereas the noise floor of the detection remains unaltered. This observation shows that phase noise affects the librational energy, without altering the free-space detection.

Finally, we study the behaviour of our system as a function of cancellation gain *g* in some more detail. To do this, we set the detuning to its optimum value *Δ* = *Ω*_*α*_ and place the particle in the anti-node of the cavity field. Figure 3c shows the measured libration occupation *n* as a function of cancellation gain *g*. At *g* = 0, despite otherwise optimal cooling conditions, excess phase noise leads to occupations above unity (*n* > 1). As *g* increases, the occupation decreases following our phase-noise model (blue area) and approaches the quantum back-action limit (black dashed line).

Figure 3d shows the rates that determine the occupation of our levitated librator under cavity-cooling as a function of the cancellation gain *g*. First, we determine the optomechanical cooling rate *γ*_opt_ as the width of the Lorentzian fit to the libration peak, shown as light-blue triangles in Fig. 3d. We observe that *γ*_opt_ does not depend on the cancellation gain *g*, as expected. Second, we extract the total heating rate *Γ* of the libration as *Γ* = *γ*_opt_*n* (ref. ^24^) and show it as red squares in Fig. 3d. The total heating rate decreases with cancellation gain *g* and approaches its fundamental limit *Γ*_BA_ (black dashed line). In the Supplementary Information, we present an independent measurement of the phase-noise heating rate performed by turning off the phase-noise cancellation and observing the population of the librator (Supplementary Information).

## Conclusions

In conclusion, we have cooled a megahertz-frequency librational mode of an anisotropic levitated nanoparticle to the quantum ground state. Cooling was provided by coupling the photons inelastically scattered by the particle to a high-finesse cavity in the resolved-sideband regime. We have used Raman sideband thermometry to determine the phonon occupation of the levitated librator. Starting at room temperature, we have achieved a minimum occupation of 0.04(1) quanta, corresponding to a state purity of 92%. A crucial technical step to reach this high purity was to minimize the impact of heating due to laser phase noise. Active phase-noise cancellation in the tweezer beam by up to ~20 dB put our system into a regime where the phonon occupation is a result of the balance between cavity-cooling and heating by radiation-torque shot noise, that is, measurement back-action. Furthermore, compared to systems cooling the centre-of-mass motion in levitation, our levitated librator operates deeper in the resolved-sideband regime due to its higher resonance frequency^40^.

The high purity achieved in our experiment places levitated librators on the forefront of experimental test beds for room-temperature quantum optomechanics experiments^27^. Furthermore, the purity of our system exceeds even that reached with gigahertz-frequency oscillators when laser-cooled in a cryogenic environment^21^. A central element setting our system apart from the canonical clamped optomechanics approach is the coherent-scattering configuration^24^. Although this configuration suffers from further back-action due to coupling of the mechanics not only to a single cavity mode but also to free space, it keeps the field strengths in the optical resonator low, circumventing excess back-action associated with thermal loading of the mirrors^27^.

In the context of multi-mode ground-state cooling of levitated systems, extending the phase-noise suppression demonstrated here to other librational and centre-of-mass modes will facilitate cavity-based six-dimensional cooling^34^ (Supplementary Information). The high-purity librational ground state could serve as a stepping stone towards preparing non-classical states of motion^31^. An interesting first step would be to squeeze the high-purity librational state by modulating the confining potential^46^, in a free fall experiment^47^ or by exploiting unstable dynamics provided by the cavity^48^. Looking further, rotational motion exhibits genuine quantum effects with no counterparts in centre-of-mass dynamics. Examples include orientational quantum revivals^47^ and quantum-persistent tennis-racket flips^49^. Finally, the megahertz motional frequency demonstrated here may open up the possibility to resonantly couple levitated nanoparticles to other well-controlled quantum systems, such as trapped atomic ions^50^, and to exploit qubit nonlinearities to engineer non-classical states of motion^51^.

## Online content

Any methods, additional references, Nature Portfolio reporting summaries, source data, extended data, supplementary information, acknowledgements, peer review information; details of author contributions and competing interests; and statements of data and code availability are available at 10.1038/s41567-025-02976-9.

## Supplementary information

Supplementary Information Supplementary Figs. 1–11, Table 1 and Discussion.

## Data Availability

Source data for Figs. 2 and 3 are available in the ETH Zurich Research Collection (www.research-collection.ethz.ch/handle/20.500.11850/738936). All other data that support the plots within this paper and other findings of this study are available from the corresponding author upon reasonable request.
