# Supplementary material for: High-purity quantum optomechanics at room temperature
Source: Nat Phys. 2025 Aug 6;21(10):1603–8. doi: 10.1038/s41567-025-02976-9 (PMC12518128; doi:10.1038/s41567-025-02976-9)
Supplement: Supplementary file 1 — Supplementary Figs. 1–11, Table 1 and Discussion. [file 41567_2025_2976_MOESM1_ESM.pdf]

nature physics

Supplementary information https://doi.org/10.1038/s41567-025-02976-9

# High-purity quantum optomechanics at room temperature

In the format provided by the authors and unedited

## CONTENTS

A: Theoretical description 1
B: Full Setup 3
C: Error in sideband thermometry from detector frequency response 4
D: particle shape characterization 5
E: Gas-dominated libration damping rates 6
F: Particle moment of inertia 7
G: Coupling strength and heating rate derivation from position scan data 7
H: Phase noise cancellation 8
I: Calibration of quasi-homodyne detection 10
J: Heating rate measurements 10
References 11

## A: THEORETICAL DESCRIPTION

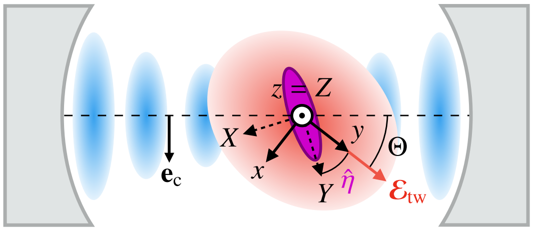


FIG. S1. Geometry of the experimental setup and coordinate systems. The scatterer's body frame is given by $(\mathbf{e}_X, \mathbf{e}_Y, \mathbf{e}_Z)$, and the laboratory frame by $(\mathbf{e}_x, \mathbf{e}_y, \mathbf{e}_z)$. In our experiment, the tweezer field $\mathcal{E}_{\rm tw}$ is polarized along the cavity axis, such that the angle $\Theta$ vanishes and the cavity field polarization $\mathbf{e}_c$ points along $x$. The operator $\hat{\eta}$ describes motion of the libration angle $\alpha$.

Here, we present the theory of librational dynamics of a nanoparticle in a levitated cavity optomechanics setup, illustrated in Fig. S1, in the presence of laser phase noise. We model the nanoparticle as a dielectric object whose polarizability tensor in the body frame $(\mathbf{e}_X, \mathbf{e}_Y, \mathbf{e}_Z)$ takes the diagonal form $(\bar{\alpha}_{\rm BF})_{ij} = \alpha_i \delta_{ij}$, where $i, j \in \{X, Y, Z\}$ and $\alpha_X < \alpha_Z < \alpha_Y$. The tweezer field, with frequency $\omega_{\rm tw}$, power $P_{\rm tw}$ and waist $W_{\rm tw}$, propagates along $\mathbf{e}_z$ and is linearly polarized along $\mathbf{e}_y$. The cavity mode has length $L_c$, waist $W_c$, a finite linewidth $\kappa$, resonant frequency $\omega_c = \omega_{\rm tw} + \Delta$ and it is polarized along direction $\mathbf{e}_c = \mathbf{e}_x \cos(\Theta) + \mathbf{e}_y \sin(\Theta)$, where $\Theta$ is the angle between the cavity axis and the polarization of the tweezer. We place the origin of coordinates at the tweezer focus and assume the particle's center of mass is fixed at that position. We also assume that the nanoparticle undergoes small-amplitude librations around its equilibrium orientation, in which the body frame $(\mathbf{e}_X, \mathbf{e}_Y, \mathbf{e}_Z)$ coincides with the laboratory one $(\mathbf{e}_x, \mathbf{e}_y, \mathbf{e}_z)$ [1]. In particular, the only degree of freedom we consider is the small angular displacement $\hat{\eta}$ in the $xy$ plane. Assuming the particle is much smaller than all relevant optical wavelengths (point-dipole approximation), the Hamiltonian reads [2]

$$\hat{H}(t) = \frac{\hat{L}^2}{2I} + \hbar\omega_c \hat{c}^\dagger \hat{c} + \hat{H}_{\rm free} - \frac{1}{2}\hat{\mathbf{E}}(\mathbf{0}, t)\bar{\alpha}_{\rm LF}(\hat{\eta})\hat{\mathbf{E}}(\mathbf{0}, t) + \hat{H}_{\rm drive}(t). \tag{S1}$$

Here, $\hat{L}$ and $I$ are angular momentum and moment of inertia of the nanoparticle, respectively, $\hat{c}$ and $\hat{c}^\dagger$ are the ladder operators of the cavity mode, and $\hat{H}_{\rm free}$ is the Hamiltonian of the free-space electromagnetic modes [3]. The fourth term in Eq. (S1) describes the optomechanical interaction, where $\bar{\alpha}_{\rm LF}(\hat{\eta})$ is the polarizability tensor of the particle in the laboratory frame and $\hat{\mathbf{E}}(\mathbf{0},t) = \boldsymbol{\mathcal{E}}_{\rm tw}(\mathbf{0},t) + \hat{\mathbf{E}}_{\rm cav}(\mathbf{0}) + \hat{\mathbf{E}}_{\rm free}(\mathbf{0})$ the total electric field at the position of the particle. This consists of (i) the classical tweezer field $\boldsymbol{\mathcal{E}}_{\rm tw}(\mathbf{0},t) = (\mathbf{e}_y/2)[E_0 e^{i\varphi(t)} e^{i\omega_{\rm tw} t} + \text{c.c.}]$, where $E_0 = \sqrt{4P_{\rm tw}/(\pi\varepsilon_0 c W_{\rm tw}^2)}$ is the field amplitude, with $\varepsilon_0$ the dielectric permittivity of vacuum and $c$ the speed of light, and $e^{i\varphi(t)}$ is a noisy phase factor whose properties we discuss below; (ii) the cavity field $\hat{\mathbf{E}}_{\rm cav}(\mathbf{0}) = \mathbf{e}_c E_c \sin(k_c y_{\rm eq})(\hat{c}^\dagger + \hat{c})$, where $y_{\rm eq}$ encodes the position of the particle along the cavity intensity profile ($k_c y_{\rm eq} = 0$ at the node, $k_c y_{\rm eq} = \pi/2$ at the antinode), and $E_c = \sqrt{\hbar\omega_c/(2\varepsilon_0 V_c)}$ is the zero-point cavity field amplitude, with $V_c = \pi(W_c/2)^2 L_c$ the mode volume of the cavity; (iii) the free-space field $\hat{\mathbf{E}}_{\rm free}(\mathbf{0})$, given in Ref. [3, Eq. (7)]. Lastly, the driving term $\hat{H}_{\rm drive}(t) = -\hbar\Omega_d(\hat{c}^\dagger + \hat{c})(e^{i\omega_{\rm tw}t} e^{i\varphi(t)} + \text{c.c.})$ models the Rayleigh scattering of tweezer photons into the cavity. The cavity driving rate $\Omega_d$ consists of two terms, $\Omega_d = \Omega_d^{\rm (id)} + \delta\Omega_d$, where $\Omega_d^{\rm (id)} = \alpha_Y E_0 E_c \sin(k_c y_{\rm eq}) \sin(\Theta)/2\hbar$ is the rate in the ideal scenario, while $\delta\Omega_d$ accounts for several uncontrolled experimental features leading to additional Rayleigh scattering (such as a small ellipticity of the tweezer polarization).

The statistical properties of $e^{i\varphi(t)}$ derive from those of the Gaussian white-noise process $\dot{\varphi}(t)$, which has first and second order moments $\langle\langle\dot{\varphi}(t)\rangle\rangle = 0$, $\langle\langle\dot{\varphi}(t)\dot{\varphi}(s)\rangle\rangle = S_{\dot{\varphi}\dot{\varphi}}\delta(t-s)$. Here, $\langle\langle\cdot\rangle\rangle$ indicates the average over all noise realizations and $S_{\dot{\varphi}\dot{\varphi}}$ is the noise power spectral density (PSD).

Assuming the particle is close to its equilibrium orientation, we proceed as follows. (1) We expand the optomechanical interaction in the limit of small librations. In doing so, we neglect four kinds of terms: (i) a term proportional to $\hat{\eta}^2(\hat{c}^\dagger + \hat{c})^2$, since it is subdominant for the parameters of this experiment, (ii) two nanoparticle-mediated coupling terms between cavity and free-space modes, which can be neglected as otherwise they would lead to a renormalization of the cavity linewidth which is not observed for subwavelength particles, (iii) two terms shifting the energy of the free-space electromagnetic modes, an effect also negligible for subwavelength particles, and (iv) a nonlinear particle-cavity coupling of the type $\hat{\eta}^2(\hat{c}^\dagger + \hat{c})$, negligible when $\Theta \approx 0$ as in our experiment. (2) We transform the resulting linear Hamiltonian to a frame rotating at the tweezer frequency $\omega_{\rm tw}$ and we undertake the rotating wave approximation (RWA) to remove terms oscillating at $2\omega_{\rm tw}$ [3]. (3) We trace out the free-space electromagnetic modes in the Born-Markov approximation, obtaining a reduced master equation for the particle-cavity dynamics. (4) We apply a displacement transformation $\hat{c} \to \hat{c} + \alpha_c(t)$, where $\alpha_c(t)$ is the classical cavity amplitude under the stochastic driving induced by phase noise, *i.e.* $\dot{\alpha}_c(t) = -(i\Delta + \kappa/2)\,\alpha_c(t) + i\Omega_d e^{-i\varphi(t)}$. At this stage, the master equation for the density operator of cavity and librational modes, $\hat{\mu}$, reads

$$\dot{\hat{\mu}}(t) = -\frac{i}{\hbar}[\hat{H}_d(t), \hat{\mu}(t)] + \frac{\kappa}{2}\mathcal{D}_{\hat{c}}[\hat{\mu}(t)] - \frac{\Gamma_{\rm BA}}{2}[\hat{q}, [\hat{q}, \hat{\mu}(t)]], \tag{S2}$$

where the Hamiltonian reads

$$\hat{H}_d(t)/\hbar = \Omega_\alpha \hat{b}^\dagger\hat{b} + \beta(t)\hat{q} + \Delta\hat{c}^\dagger\hat{c} + G\hat{q}(\hat{c}e^{i\varphi(t)} + \hat{c}^\dagger e^{-i\varphi(t)}). \tag{S3}$$

In the above equations, we introduced the quadrature $\hat{q} = \hat{\eta}/\alpha_{\rm zpf} = \hat{b}^\dagger + \hat{b}$ where $\hat{b}$, $\hat{b}^\dagger$ are the ladder operators of the librational mode and $\alpha_{\rm zpf} = \sqrt{\hbar/(2I\Omega_\alpha)}$ is the mechanical zero-point angular displacement, the librational frequency $\Omega_\alpha = \sqrt{(\Delta\alpha)E_0^2/2I}$, the coupling strength

$$G = -(\Delta\alpha)\alpha_{\rm zpf}E_0 E_c \sin(k_c y_{\rm eq})\cos(\Theta)/(2\hbar), \tag{S4}$$

and the stochastic mechanical driving $\beta(t) = 2G\Re\left[e^{i\varphi(t)}\alpha_c(t)\right]$, as well as $\Delta\alpha = \alpha_Y - \alpha_X$. Eq. (S2) also includes a Lindblad dissipator for the decay of the cavity mode ($\mathcal{D}_{\hat{a}}[\hat{\mu}] = \hat{a}\hat{\mu}\hat{a}^\dagger - \frac{1}{2}\{\hat{a}^\dagger\hat{a}, \hat{\mu}\}$) and a position-localization dissipator representing photon recoil heating (shot noise), with rate

$$\Gamma_{\rm BA} = \frac{(\Delta\alpha)^2 E_0^2 \alpha_{\rm zpf}^2 \omega_{\rm tw}^3}{(12\pi\hbar c^3\varepsilon_0)}. \tag{S5}$$

One can show that the noisy multiplicative phase factor in the last term of Eq. (S3) is negligible, $e^{i\varphi(t)} \approx 1$, under the assumption $S_{\dot{\varphi}\dot{\varphi}} \ll \kappa$, which is the regime of our experiment. Hence, the main effect of phase noise is a stochastic force proportional to $\beta(t)$, which leads to additional heating of the particle's motion.

To explore the librational dynamics we adiabatically eliminate the cavity mode assuming $G \ll \kappa$ [4, 5] and we average over all possible realizations of the noise following the second-order generalized cumulant expansion method [6, 7]. After applying a final displacement transformation $\hat{b} \to \hat{b} + \lim_{t\to\infty}\langle\langle\beta(t)\rangle\rangle$, the master equation for the density operator of the librations, $\hat{\rho}$, reads

$$\dot{\hat{\rho}}(t) = -i[\Omega_\alpha\hat{b}^\dagger\hat{b}, \hat{\rho}(t)] - \frac{1}{2}(\Gamma_{\rm BA} + \Gamma^{(\varphi)})[\hat{q}, [\hat{q}, \hat{\rho}(t)]] + \Gamma_+^{(c)}\mathcal{D}_{\hat{b}^\dagger}[\hat{\rho}(t)] + \Gamma_-^{(c)}\mathcal{D}_{\hat{b}}[\hat{\rho}(t)]. \tag{S6}$$

The adiabatic elimination of the cavity leads to the Lindbladians for heating and cooling with rates $\Gamma_{\pm}^{(c)} = G^2\kappa/[(\Omega_\alpha \pm \Delta)^2 + (\kappa/2)^2]$, as well as a small shift of the libration frequency which can be neglected for this experiment. Since in our experiment $\Gamma_{+}^{(c)} \ll \Gamma_{-}^{(c)}$, the total cooling rate $\gamma_{\rm opt} = \Gamma_{+}^{(c)} - \Gamma_{-}^{(c)}$ can be approximated as

$$\gamma_{\rm opt} \approx -\Gamma_{-}^{(c)} = -\frac{G^2\kappa}{(\Omega_\alpha - \Delta)^2 + \left(\frac{\kappa}{2}\right)^2}. \tag{S7}$$

Note that phase noise induces an additional heating term with rate $\Gamma_\varphi$, which in the weak noise limit $S_{\dot\varphi\dot\varphi} \ll \kappa$ reads

$$\Gamma^{(\varphi)} \approx 4G^2 n_{\rm cav} S_{\dot\varphi\dot\varphi} \frac{[\left(\frac{\kappa}{2}\right)^2 - \Delta^2]^2 + \left(\Omega_\alpha \frac{\kappa}{2}\right)^2}{[\Delta^2 + \left(\frac{\kappa}{2}\right)^2][(\Delta + \Omega_\alpha)^2 + \left(\frac{\kappa}{2}\right)^2][(\Delta - \Omega_\alpha)^2 + \left(\frac{\kappa}{2}\right)^2]}, \tag{S8}$$

where $n_{\rm cav} = \lim_{t\to\infty}\langle\langle|\alpha_c(t)|^2\rangle\rangle \approx \Omega_d^2/[\Delta^2 + (\kappa/2)^2]$ is the steady-state cavity occupation in the weak noise limit. As a remark, the phase noise-induced heating rate increases as $\Gamma^{(\varphi)} \propto G^2 n_{\rm cav} \propto \sin^4(k_c y_{\rm eq})$ as the particle moves from the node to the antinode of the cavity intensity profile. In Eq. (S6) we neglect any dissipation due to collisions with gas molecules, as they become negligible at the experiment's pressure $P_{\rm gas} = 5 \times 10^{-9}$ mbar [8].

From Eq. (S6) we can compute the steady-state number of librational phonons as $n = \lim_{t\to\infty} {\rm tr}\{\hat b^\dagger \hat b \hat\rho(t)\} = -(\Gamma_{\rm BA} + \Gamma^{(\varphi)})/\gamma_{\rm opt}$, where we neglected the cavity contribution to the total heating rate since $\Gamma_{+}^{(c)} \ll \Gamma_{\rm BA}$. This leads to the expression

$$n = n^{(0)} + n^{(\varphi)} = \frac{\Gamma_{\rm BA}[(\Delta - \Omega_\alpha)^2 + \left(\frac{\kappa}{2}\right)^2]}{G^2\kappa} + 4n_{\rm cav} S_{\dot\varphi\dot\varphi} \frac{[\left(\frac{\kappa}{2}\right)^2 - \Delta^2]^2 + \left(\Omega_\alpha \frac{\kappa}{2}\right)^2}{[\Delta^2 + \left(\frac{\kappa}{2}\right)^2][(\Delta + \Omega_\alpha)^2 + \left(\frac{\kappa}{2}\right)^2]\kappa}, \tag{S9}$$

which has been used to fit the experimental data. The two terms on the right-hand side of Eq. (S9) are respectively the phonon number in the absence of phase noise, $n^{(0)}$, and the contribution due to phase noise, $n^{(\varphi)}$. In the regime of weak noise ($S_{\dot\varphi\dot\varphi} \ll \kappa$), resolved sidebands ($\kappa \ll \Omega_\alpha$) and optimal detuning ($\Delta = \Omega_\alpha$), $n^{(\varphi)}$ takes the approximate form $n^{(\varphi)} \approx n_{\rm cav} S_{\dot\varphi\dot\varphi}/\kappa$, in agreement with the expressions known in literature [9–11].

## B: FULL SETUP

A sketch of the experimental setup is shown in Fig. S2. We load a particle into vacuum with a load-lock system [12]. After loading at around $1 \times 10^{-2}$ mbar we evacuate the chamber to below $1 \times 10^{-8}$ mbar using an ion-getter pump and position the particle along the cavity standing wave by slightly shifting the trapping lens mounted to a nanopositioner stage (not drawn). The polarization of the main beam generating the tweezer can be adjusted with a half-wave plate (HWP) and a quarter-wave plate (QWP) in front of the vacuum chamber. We lock the cavity length to the laser wavelength by adjusting the position of mirror A mounted to a piezo stage. The necessary lock-beam is picked off by a beam splitter from the main laser beam. We frequency shift it via an acousto optic modulator (AOM1) and an electro optic modulator (EOM1) to match the frequency of the transverse electromagnetic mode $\mathrm{TEM}_{10}$ of the cavity. In this way, no additional light at the frequency of the $\mathrm{TEM}_{00}$ mode is introduced into the cavity that could cause heating of the particle, and we instead lock the cavity to the $\mathrm{TEM}_{10}$ mode. We add 24.5 MHz sidebands to this lock beam and inject it into the cavity. The reflection signal is detected on a fast photodiode ($\mathrm{PD}_{\mathrm{PDH}}$) and used to generate the error signal for a PID controller adjusting the piezo voltage, and thus the cavity length.

We detect the motion of our particle by collecting the back-scattered light. From AOM1 and AOM2 we derive a local oscillator beam at $\omega_{\rm LO}$. It interferes with the back-scattered light from the particle and is distributed equally onto two balanced photodiodes through a fiber-based polarization controller and polarizing beam splitter. We present power spectral densities of this balanced heterodyne detection, frequency shifted to $\omega_{\rm LO}$, throughout this work and in Fig. S3. We can identify all six motional degrees of freedom of the particle in the spectrum, which was recorded at $1 \times 10^{-5}$ mbar with the particle positioned close to the anti-node of the cavity. The center-of-mass motion peaks of the transverse axes $x$ and $y$ are weakly cooled and show up as sharp peaks around 250 kHz, while the $z$ motion around 80 kHz is broadened due to cavity cooling at the anti-node [13]. The low frequency $\gamma$ libration peak can be found among the center of mass peaks, corresponding to the weak confinement of particle orientation along its long axis (in the $zy$ plane). The other two modes representing librations around the equilibrium alignment of the long particle axis with the laser polarization are found at frequencies much larger than the center-of-mass frequencies. In this work, we focus on the highest frequency $\alpha$ mode around 1.1 MHz, which is oscillating in the $xy$ plane. The noise floor in the spectrum is given by shot noise from the local oscillator.

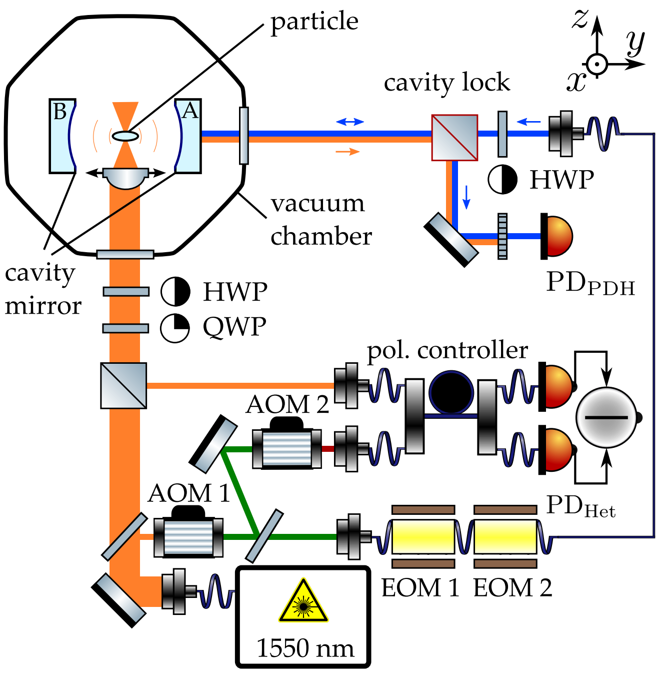


FIG. S2. **Full setup for trapping, cavity lock and detection.** The particle is levitated inside the optical cavity (cavity mirrors labeled A and B, respectively), which is situated within the vacuum chamber. The polarization of the main tweezer beam is controlled by a half-wave plate (HWP) and a quarter-wave plate (QWP). Light scattered backwards by the particle is reflected by the polarizing beam splitter (black outline). This signal is then interfered with a local oscillator, which we derive from the main beam and frequency shift it to $\omega_{\rm LO}$ via two acousto-optic modulators (AOM1 and AOM2). Two balanced photodiodes, receiving half of the combined light each, provide the difference signal for the balanced heterodyne detection. For the cavity lock, we derive a beam at a frequency close to the difference frequencies of transverse electromagnetic modes $\rm TEM_{10}$ and $\rm TEM_{00}$ with AOM1 and EOM1. We add 24.5 MHz sidebands to this beam with EOM2, cross polarize it to the tweezer with a HWP and send it on the back side of cavity mirror A. The reflected signal is directed into a fast photodiode ($\rm PD_{PDH}$) via a beam splitter (red outline, 90% reflection) and a polarizer, which suppresses light scattered by the particle. The output of $\rm PD_{PDH}$ is used to generate an error signal and lock the cavity.

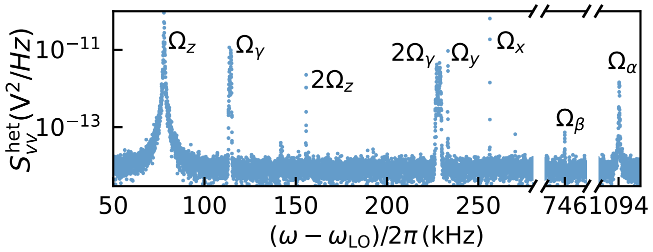


FIG. S3. **Heterodyne spectrum of all six motional modes.** Our heterodyne detection of back-scattered light is sensitive to three COM modes at $\Omega_z$ (along the tweezer propagation), $\Omega_y$ (along the tweezer polarization, aligned with the cavity axis) and $\Omega_x$, as well as three librational modes at $\Omega_\alpha$ (in the $xy$ plane), $\Omega_\beta$ (in the $yz$ plane) and $\Omega_\gamma$ (in the $xz$ plane). We observe second harmonics at $2\Omega_z$ and $2\Omega_\gamma$ due to the modes' large motional amplitudes exploring nonlinearities in their respective confining potentials at a pressure of $10^{-5}$ mbar, where this spectrum has been taken and all modes are well visible due to their thermal population. In the main text, we only focus on the highest frequency libration mode at $\Omega_\alpha$.

## C: ERROR IN SIDEBAND THERMOMETRY FROM DETECTOR FREQUENCY RESPONSE

The occupation numbers presented in the main text are extracted from sideband asymmetry measurements. A correct estimation of the asymmetry relies on a frequency-independent response of the acquisition chain composed by the photodetector and the data acquisition system [14]. Here we present an analysis on the frequency response of the detection at the libration frequency $\Omega_\alpha/(2\pi)$, and show a negligible contribution of the detector response to the librational occupations.

The libration occupation number inferred from the sideband asymmetry is extracted according to $n_{\rm inf} = \frac{a}{1-a}$, where $a$ is the

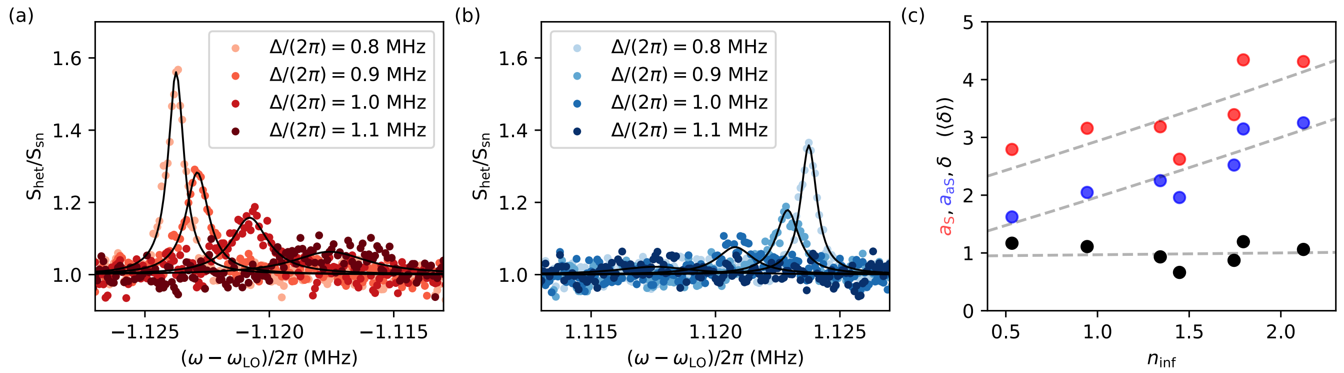


FIG. S4. **Sideband asymmetry consistency.** Heterodyne spectra $S_{\mathrm{het}}$ near the (a) Stokes and (b) anti-Stokes sidebands for different cavity detunings $\Delta$. The same spectra are shown in the main text in Fig. 2(b) with adjusted frequency axes. We extract the power carried by each sideband as $a_{\mathrm{S}}$ for the Stokes and $a_{\mathrm{aS}}$ for the anti-Stokes sidebands from Lorentzian fits, shown here as black lines. (c) Stokes (red circles) and anti-Stokes (blue circles) sidebands power normalized by their average difference $\langle\delta\rangle$ as function of the inferred occupation number $n_{\mathrm{inf}}$. The Stokes and anti-Stokes data points are up-shifted by one for clarity. The dashed lines are fits to the model of Eq. (S10) that accounts for a frequency-dependent response function in the heterodyne detection.

measured sideband asymmetry. A detection with a frequency-dependent response records an asymmetry $a = c_+ n/(c_-(n+1))$, where $c_-$ and $c_+$ are the detection responsivity factors at the Stokes and anti-Stokes frequencies, respectively, and where $n$ represents the true occupation number. By plugging the modified asymmetry $a$ in the formula for $n_{\mathrm{inf}}$ and inverting for $n$, $n+1$ and their detected differences, respectively, we get

$$\begin{aligned} n &= \frac{n_{\mathrm{inf}}}{\frac{c_-}{c_+}(n_{\mathrm{inf}}+1) - n_{\mathrm{inf}}}, \\ n+1 &= \frac{\frac{c_-}{c_+}(n_{\mathrm{inf}}+1)}{\frac{c_-}{c_+}(n_{\mathrm{inf}}+1) - n_{\mathrm{inf}}}, \text{ and} \\ c_-(n+1) - c_+ n &= \frac{c_-}{\frac{c_-}{c_+}(n_{\mathrm{inf}}+1) - n_{\mathrm{inf}}}. \end{aligned} \tag{S10}$$

The equations above relate the inferred occupation $n_{\mathrm{inf}}$ and the true occupation $n$ via the response function of the measurement apparatus. For an ideal detector with a flat frequency response $c_+ = c_-$, and from the above formula we get $n = n_{\mathrm{inf}}$. For a real detector, we have $c_+ \neq c_-$, and the inferred occupation deviates from the true one. Note that the power carried by the anti-Stokes sideband $a_{\mathrm{aS}}$ is proportional to the true occupation number $a_{\mathrm{aS}} \propto n$, while the power carried by the Stokes sideband $a_{\mathrm{S}}$ is proportional to the true occupation number plus one $a_{\mathrm{S}} \propto (n+1)$. To quantify the infidelity of our occupation estimation, we have collected data for $n_{\mathrm{inf}}$, $a_{\mathrm{S}}$ and $a_{\mathrm{aS}}$, and simultaneously fit them to Eq. (S10), to extract $c_-/c_+$.

Figures S4(a) and (b) show heterodyne spectra with the Stokes and anti-Stokes librational peaks, respectively, for different values of the cavity detuning $\Delta$. These spectra are the same ones shown in the main text in Fig. 2(b). We observe the peaks shrinking in size due to the effect of cavity cooling when approaching the optimal detuning. Furthermore, the frequency of the libration mode slightly shifts due to cavity coupling, an effect also observed for cooling of center-of-mass modes [12]. We extract $a_{\mathrm{S}}$ and $a_{\mathrm{aS}}$ from Lorentzian fits shown as black lines. Figure S4(c) shows their values and difference $\delta$ plotted as a function of the libration occupation inferred via sideband asymmetry $n_{\mathrm{inf}}$. The dashed lines are fits to Eq. (S10), from which we extract a measurement chain asymmetry $c_-/c_+ = 0.97(5)$. The inferred ground state occupation of $n_{\mathrm{inf}} = 0.5(3)$ underestimates the true one by a factor of 1.04. This systematic error is much smaller than the error from the Lorentzian fits used to infer the occupation.

## D: PARTICLE SHAPE CHARACTERIZATION

The center-of-mass damping rates in the gas-dominated regime give insights on the trapped particle shape. In this regime, the gas damping rates depend on the effective particle cross section for each direction of COM motion, resulting in three different damping values for anisotropic particles [15].

We have measured the COM damping rates of the particle at high pressure ($\sim$ mbar) and in the absence of cavity cooling ($\Delta \gg \Omega_\alpha$). For these measurements, the tweezer is linearly polarized along the $y$ axis. This results in all three orientation angles of the particle being harmonically trapped.

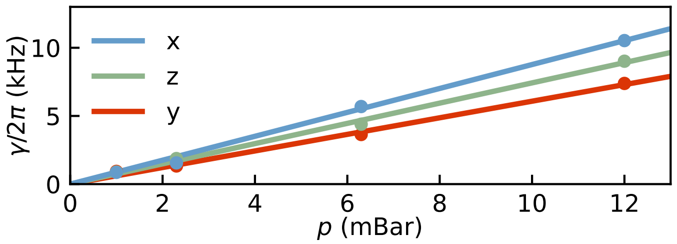


FIG. S5. **Gas-induced damping rates** We extract the gas damping rates from the mechanical linewidths of the three center-of-mass modes in the heterodyne spectra at high pressures. Lines are linear fits to the damping rates and the fractions of their slopes give an estimate of the particle shape.

We fit a Lorentzian lineshape to each center-of-mass peak in the PSD and extract the corresponding damping rate as the width. The measured COM damping rates as a function of pressure are presented in Fig. S5.

From the linear fits we extract the slopes $\bar{\gamma}_x/(2\pi) = 0.88(2)\,\mathrm{kHz\,mbar^{-1}}$, $\bar{\gamma}_y/(2\pi) = 0.61(2)\,\mathrm{kHz\,mbar^{-1}}$ and $\bar{\gamma}_z/(2\pi) = 0.74(2)\,\mathrm{kHz\,mbar^{-1}}$, which results in fractions of $(\gamma_\mathrm{x}, \gamma_\mathrm{y}, \gamma_\mathrm{z})/\gamma_\mathrm{y} = (1.4, 1, 1.2)$ for the first particle used in our experiments. An identical analysis for the second particle yields the ratios $(\gamma_\mathrm{x}, \gamma_\mathrm{y}, \gamma_\mathrm{z})/\gamma_\mathrm{y} = (1.5, 1, 1.05)$. The fact that we observe the lowest damping rate along the polarization direction of the tweezer ($y$ axis) indicates that the particle's long axis aligns with the field polarization, as expected. The difference in the three damping rates further indicates that the particles shape is three-dimensionally anisotropic, which is commensurate with the observation of three distinct libration modes.

## E: GAS-DOMINATED LIBRATION DAMPING RATES

Here we provide measurements of the damping rate of the $\alpha$ libration mode for several values of pressures. Figure S6(c) shows a spectrum of the libration detector obtained at a pressure of 6.3 mbar, where the peak of the $\alpha$ mode has been fitted to a Lorentzian function to deduce the damping rate. For this and the following measurements, the cavity cooling was turned off in order to not provide additional optical damping. Similar to the center-of-mass modes, the damping rate of the $\alpha$ mode is linearly dependent on

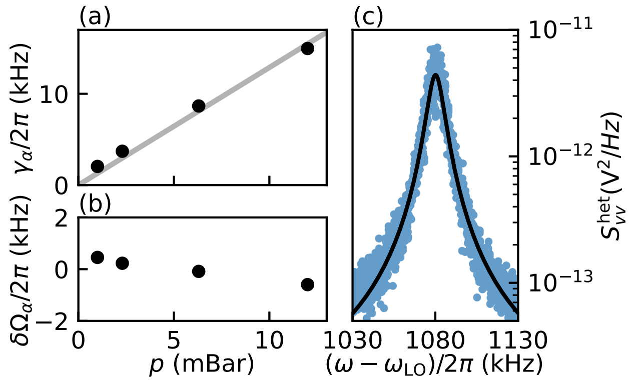


FIG. S6. **Pressure dependence of linewidth and frequency of the $\alpha$ libration mode.** (a) Similar to the center-of-mass modes, we fit (gray) the pressure dependence of the libration damping rate. (b) The difference $\delta\Omega_\alpha = \Omega_\alpha(p) - \bar{\Omega}_\alpha$ of the $\alpha$ mode frequency from the mean $\bar{\Omega}_\alpha$ remains below 0.1% for our pressure range. (c) Lorentzian fit to extract $\gamma_\alpha$ in (a) and $\Omega_\alpha$ in (b) at a pressure of $p = 6.3$ mbar.

pressure[Fig. S6(a)], while its frequency does not depend on pressure [Fig. S6(b)]. By linear extrapolation from the fit in Fig. S6(a), we can estimate the damping rate for the pressures in the experiments of the main text as $\gamma_\alpha(5 \times 10^{-9}\,\mathrm{mbar}) \approx 2\pi \times 6.6 \times 10^{-6}\,\mathrm{Hz}$. With the value of $\gamma_\alpha$ at this low pressure, we estimate the thermal heating rate $\Gamma_\mathrm{gas} = \gamma_\alpha k_\mathrm{B} T/(\hbar\Omega_\alpha) = 2\pi \times 40\,\mathrm{Hz}$.

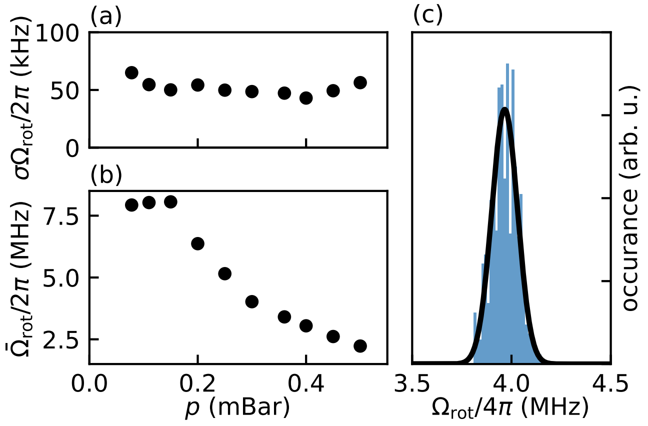


FIG. S7. **Pressure dependence of rotation frequency fluctuations and central rotation frequency of spinning particle.** (a) The standard deviation $\sigma_{\Omega_{\rm rot}}$ of the measured rotation rates remains largely constant over our pressure range. (b) The center frequency shows a pressure dependence characteristic for rotation, as the particle spins faster at lower pressures. (c) Histogram of the rotation rate at 0.08 mbar with a Gaussian fit to extract the standard deviation $\sigma_{\Omega_{\rm rot}}$ in (a) and central frequency $\bar{\Omega}_{\rm rot}$ in (b).

## F: PARTICLE MOMENT OF INERTIA

We measure the particle's moment of inertia by studying the behavior in rotation. The libration motion transitions to rotation when the particle is driven with circularly polarized light. We observe this transition in our spectra as we turn the quarter-wave plate in front of our vacuum chamber (see Fig. S2). We can discriminate rotational motion against center-of-mass and libration motion by its frequency changing with pressure. The rotation frequency of a spinning particle shows thermal fluctuations [16]. Figure S7(c) displays a histogram of the measured rotation frequency. From a Gaussian fit to the histogram, we extract the standard deviation $\sigma_{\Omega_{\rm rot}}$ and central frequency in Figs. S7 (a) and (b), respectively. While the standard deviation remains constant, the central frequency increases as the pressure is lowered. From the standard deviation, we can infer the moment of inertia associated with the $\alpha$-mode of the particle via [16]

$$\sigma_{\Omega_{\rm rot}} = \frac{1}{2\pi}\sqrt{\frac{k_B T}{I}}\,. \tag{S11}$$

With our measured $\sigma_{\Omega_{\rm rot}} = 52(6)$ kHz at $T \approx 300$ K we estimate $I = 3.9(9) \times 10^{-32}$ kg m$^2$. An identical analysis has been carried out for the second particle used to collect the data including the phase noise cancellation, resulting in $I = 1.3(4) \times 10^{-32}$ kg m$^2$.

## G: COUPLING STRENGTH AND HEATING RATE DERIVATION FROM POSITION SCAN DATA

In this section we detail how the cavity coupling strength and the heating rates have been derived from our measurements. We extract the cavity coupling strength $G$ from the measured cooling rate $\gamma_{\rm opt}$. Figure S8 shows the measured cooling rates $\gamma_{\rm opt}$, obtained as the linewidth of the librational Lorentzian peaks, for several particle positions $y_{\rm eq}$. This experiment was done at the optimal cavity detuning $\Delta = \Omega_\alpha$, and at a pressure of $5 \times 10^{-9}$ mbar. Both these conditions ensure that the librational damping is dominated solely by cavity cooling, and not by the residual gas.

From a fit of the measured $\gamma_{\rm opt}(y_{\rm eq})$ to its theory expression derived in Eq. (S7), we extract a cavity coupling strength $G/(2\pi) = 46.9(2)\,\text{kHz} \times \sin(ky_{\rm eq})$ and $31.5(3)\,\text{kHz} \times \sin(ky_{\rm eq})$ for the first and second particle, respectively. In the fit, the coupling strength is the only free parameter, while the values of the cavity linewidth and detuning, and the libration frequency have been independently measured. By comparison, the theoretical values of the coupling rate, which are shown in Table I, are $G/(2\pi) = 35(4)\,\text{kHz} \times \sin(ky_{\rm eq})$ and $19(3)\,\text{kHz} \times \sin(ky_{\rm eq})$.

The total libration heating rate $\Gamma = \Gamma_{\rm BA} + \Gamma^{(\varphi)}$ is extracted from the data of occupation $n$ as function of position $y_{\rm eq}$ shown in the main text in Fig. 3(a). We fit the measured values of $n$ to the formula for steady-state cavity cooling derived in Eq. (S9), with $\Gamma_{\rm BA}$ and the number of cavity photons $n_{\rm cav}$ as the only free parameters. From the fit, we obtain the values $\Gamma_{\rm BA}/(2\pi) = 0.7(1)$ kHz and $n_{\rm cav} = 1.9(4) \times 10^6 \times \sin^2(ky_{\rm eq})$ for the first particle, and $\Gamma_{\rm BA}/(2\pi) = 0.5(1)$ kHz and $n_{\rm cav} = 6.8(2) \times 10^6 \times \sin^2(ky_{\rm eq})$ for the second one. Note that for this fit we used the value of $G$ obtained from the cooling rate data with the procedure outlined above, and a value of the laser phase noise of $\sqrt{S_{\dot\phi\dot\phi}(\Omega_\alpha)}/(2\pi) = 0.16(3)\,\text{Hz}/\sqrt{\text{Hz}}$ which we have directly measured by using the optical

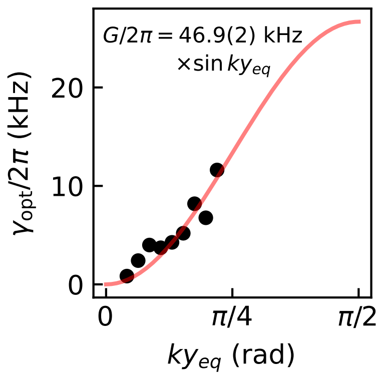


FIG. S8. **Cavity coupling strength**. Libration linewidth $\gamma_{\mathrm{opt}}$ (black dots) measured for different particle positions $y_{\mathrm{eq}}$ across the cavity standing wave. The red line is a fit to Eq. (S7), from which we extract a libration-cavity coupling strength $G/(2\pi) = 46.9(2)\,\mathrm{kHz} \times \sin(ky_{\mathrm{eq}})$. These measurements were done at optimal cavity detuning $\Delta \approx \Omega_\alpha$ and at a pressure of $5 \times 10^{-9}$ mbar, where the librational linewidth is fully dominated by cavity cooling and $\gamma_{\mathrm{opt}}$ represents the optical cooling rate.

| Symbol | Explanation | Particle 1 | Particle 2 |
|---|---|---|---|
| $\Omega_\alpha/(2\pi)$ | Libration frequency | 1.1 MHz | 1.1 MHz |
| $W_{\mathrm{tw}}$ | Tweezer beam waist | 0.85 µm | 0.85 µm |
| $W_c$ | Cavity waist | 48 µm | 48 µm |
| $L_c$ | Cavity length | 6.4 mm | 6.4 mm |
| $E_0$ | Tweezer field amplitude $\sqrt{4P_{\mathrm{tw}}/(\pi\varepsilon_0 c W_{\mathrm{tw}}^2)}$ | $2.8 \times 10^7$ V/m | $2.8 \times 10^7$ V/m |
| $E_c$ | Zero-point cavity field amplitude $\sqrt{\hbar\omega_c/(2\varepsilon_0\pi(W_c/2)^2 L_c)}$ | 24.3 V/m | 24.3 V/m |
| $I$ | Moment of inertia | $3.9 \times 10^{-32}$ kg m$^2$ | $1.3 \times 10^{-32}$ kg m$^2$ |
| $\alpha_{\mathrm{zpf}}$ | Zero-point angular displacement $\sqrt{\hbar/(2I\Omega_\alpha)}$ | 14 µrad | 24 µrad |
| $\Delta\alpha$ | Difference in polarizability of the long and short axes $2I\Omega_\alpha^2/E_0^2$ | $4.7 \times 10^{-33}$ Cm$^2$/V | $1.5 \times 10^{-33}$ Cm$^2$/V |
| $G/(2\pi)$ | Libration cavity coupling $(\Delta\alpha)\alpha_{\mathrm{zpf}} E_0 E_c/(4\pi\hbar)$ | 35 kHz (theory), 47 kHz (fit) | 19 kHz (theory), 31 kHz (fit) |
| $\Gamma_{\mathrm{BA}}/(2\pi)$ | Tweezer photon recoil heating rate $\frac{(\Delta\alpha)^2 E_0^2 \alpha_{\mathrm{zpf}}^2 \omega_{\mathrm{tw}}^3}{(24\pi^2\hbar c^3\varepsilon_0)}$ | 1.0 kHz (theory), 0.7 kHz (fit) | 0.3 kHz (theory), 0.5 kHz (fit) |

TABLE I. **Theory parameters and their values for each particle presented in this work.**

setup described in Fig. S9(a). By comparison, the theory predicts a photon recoil heating rates of $\Gamma_{\mathrm{BA}}^{\mathrm{th}}/(2\pi) = 1.0(2)\,\mathrm{kHz}$ and $\Gamma_{\mathrm{BA}}^{\mathrm{th}}/(2\pi) = 0.3(1)\,\mathrm{kHz}$ for each particle, respectively.

The theory curves shown together with the data in Fig. 2(c) and Fig. 3(a) are calculated by plugging into Eq. (S9) the values of heating rate and coupling strength derived from the fits, and the values of cavity linewidth, detuning, and libration frequency from independent measurements. The thickness of the theory lines are 1-sigma confidence intervals of the fitted values.

## H: PHASE NOISE CANCELLATION

In this section, we detail the method employed to cancel laser phase noise. In our setup, a single laser is used for trapping and cooling the nanoparticle via cavity cooling. The laser has a noisy frequency $\omega_{\mathrm{TW}} + \delta\omega$, where $\omega_{\mathrm{TW}}$ is the carrier tweezer frequency and $\delta\omega$ represents its fluctuations. The frequency fluctuations $\delta\omega$ have a power spectral density $S_{\omega\omega}(\Omega)$, where $\Omega$ is the Fourier frequency of the noise. As derived in Sec. A of this supplement, only noise components in a narrow bandwidth around the frequency $\Omega = \Omega_\alpha \sim 2\pi \times 1\,\mathrm{MHz}$ contribute to heating up the libration motion. Therefore, to suppress this excess heating rate, we realize a noise eater to reduce the value of $S_{\omega\omega}(\Omega_\alpha)$. Note that frequency and phase are linked by $\omega = \dot{\phi}$, consequently their fluctuations follow the spectral relation $S_{\omega\omega}(\Omega) = S_{\dot{\phi}\dot{\phi}}(\Omega) = \Omega^2 S_{\phi\phi}(\Omega)$, so for our purpose there is no distinction between phase and frequency noise, apart from a multiplicative factor.

Following the approach of Ref. [17], we build a phase noise eater composed of an imbalanced interferometer to measure laser phase noise, and a real-time feedback controller to suppress noise components in a narrow band around 1 MHz. The experimental setup is presented in Fig. S9(a). Before laser light arrives to the trapping setup, a small portion of the beam (10 mW) is collected and sent to a fiber path-imbalanced Mach-Zehnder interferometer: light entering the interferometer is split into two paths by a 50:50 fiber beam splitter. The long arm of the interferometer introduces a delay $\tau = Ln_f/c$, where $L$ is the fiber length and $n_f$ is its

refractive index. After the delay stage, the two paths recombine at another 50:50 beam splitter, and the output light intensity is measured with a balanced photo-detector (BPD). For an input laser beam with a fluctuating phase $\phi(t)$, the output phase at the BPD is proportional to $\phi(t) - \phi(t-\tau)$. Therefore, the interferometer's transfer function $R(\Omega)$ is expressed as

$$R(\Omega) = 1 - e^{-i\tau\Omega}. \tag{S12}$$

The fiber length determines the frequencies at which the interferometer exhibits maximum sensitivity. To optimize sensitivity at a specific frequency $\Omega_\phi$, the delay length required is $L = c\pi/n_f\Omega_\phi$. We use a delay fiber of 80 m, which gives us an optimal interferometric sensitivity at a frequency of $\sim$ 1 MHz. To stabilize the interferometer against environmental thermal and acoustic noise, we enclose it into a shielded box. Furthermore, we wrap the whole delay fiber around a home-built fiber stretcher to be able to control the slowly-varying relative phase between the two interferometer arms.

In order to get a real-time measurement of laser phase noise, the interferometer needs to be locked. The BPD output is directed to a FPGA card that implements a low-pass filter (cut-off frequency of 10 Hz) and a PID controller to obtain the control signal. This control signal gets then amplified and sent to the piezo of the fiber stretcher. Thanks to the lock and the shielding box, the interferometer remains stable for hours.

Once we have an efficient and stable laser phase-noise measurement, we implement feedback to the laser phase to suppress noise at frequencies around $\Omega_\alpha$. The feedback actuator is a fiber electro-optic modulator (EOM) mounted in between the seed and the laser amplifier (see Fig. S9(a)). To generate the feedback signal, we process the BPD signal with the FPGA, which implements an I/Q demodulation. The controller transfer function reads

$$H(\Omega) = g e^{-i\Omega\tau_{\mathrm{IQ}}} \frac{\gamma_{\mathrm{IQ}}\Omega}{\Omega_{\mathrm{IQ}}^2 - \Omega^2 + i\gamma_{\mathrm{IQ}}\Omega}, \tag{S13}$$

where $\gamma_{\mathrm{IQ}}$ is the linewidth for noise suppression, $\Omega_{\mathrm{IQ}} = \Omega_\alpha$ the central frequency where cancellation is strongest, $g$ is the cancellation gain that we also mention in the main text, and $\tau_{\mathrm{IQ}}$ is a tunable delay term used to compensate for delays in the setup. With the feedback transfer function $H$ in our hands, we can derive an expression for the laser phase-noise spectrum under feedback $S^{\mathrm{fb}}_{\dot\phi\dot\phi}$:

$$S^{\mathrm{fb}}_{\dot\phi\dot\phi}(\Omega) = \frac{S_{\dot\phi\dot\phi}(\Omega)}{|1 + M(\Omega)R(\Omega)H(\Omega)|^2}, \tag{S14}$$

where $S^{\mathrm{fb}}_{\dot\phi\dot\phi}(\Omega)$ is laser phase noise in the absence of feedback, and $M(\Omega)$ is the transfer function of the phase EOM. From the measured spectra $S^{\phi}_{VV}$ of the BPD's output, $S_{\dot\phi\dot\phi}$ can be expressed as

$$S_{\dot\phi\dot\phi}(\Omega) = \frac{C^2_{\phi V}\Omega^2 S^{\phi}_{VV}(\Omega)}{|R(\Omega)|^2}, \tag{S15}$$

where $C_{\phi V}$ is the calibration factor to transform voltage fluctuations measured at BPD into laser phase fluctuations. We have determined $C_{\phi V}$ by imparting a known harmonic modulation to the laser phase via the EOM, and measuring the resulting signal at BPD. The term $\Omega^2$ in Eq. (S15) accounts for the conversion from phase noise $S_{\phi\phi}$ to frequency noise $S_{\dot\phi\dot\phi}$.

In Fig. S9(b), phase noise spectra under active cancellation are shown. By increasing the gain from $g = 0$ (no feedback) up to $g = 1.0$, a suppression of 20 dB is reached at $\Omega = \Omega_\alpha$. This suppression is an order of magnitude higher than that reported by [17], which we attribute to the reduced impact of fiber noise at our operating wavelength. In the inset, by changing the cancellation $g$, we verified that $S_{\dot\phi\dot\phi}$ follows the expected trend, which for $\Omega_\alpha = \Omega_{\mathrm{IQ}}$ is given by

$$S^{\mathrm{fb}}_{\dot\phi\dot\phi}(\Omega_\alpha) = \frac{S_{\dot\phi\dot\phi}(\Omega_\alpha)}{|1 + gM(\Omega_\alpha)R(\Omega_\alpha)|^2}. \tag{S16}$$

The implemented scheme can be extended for multi-mode phase-noise cancellation. This is achieved by modifying the control law in Eq. (S13) to

$$H(\Omega) = \sum_{j=0}^{m} g_i e^{-i\Omega\tau_{\mathrm{IQ,j}}} \frac{\gamma_{\mathrm{IQ,j}}\Omega}{\Omega_{\mathrm{IQ,j}}^2 - \Omega^2 + i\gamma_{\mathrm{IQ,j}}\Omega}, \tag{S17}$$

resulting in $m$ individual filters, that can be tuned independently. The suppression factor remains constrained by the sensitivity of the interferometer, which is optimized for a specific frequency. Assembling individual interferometers, each connected to an FPGA, would allow optimal detection for each of the target modes.

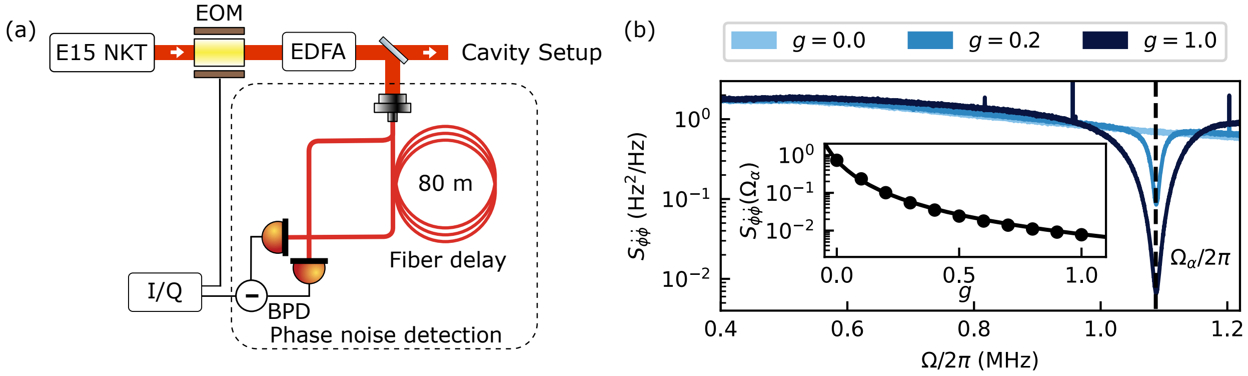


FIG. S9. **Phase noise cancellation setup.** (a) Experimental setup for phase noise measurement and cancellation. A 1550 nm laser (E15 NKT Photonics) outputs 30 mW of laser power, which is directly sent to a phase EOM. The modulated beam is then amplified to 2 W using an Erbium-doped Fiber Amplifier (EDFA). A small portion of this power (10 mW) is tapped and sent to a path-imbalanced Mach-Zender interferometer: the light is launched into a single-mode fiber (SMF) and split into two beams by a fiber-based 50:50 beam splitter (BS). After the BS, the light traveling in one of the two arms is delayed by a ~ 80 m SMF to enhance the phase noise sensitivity at a frequency of ~1 MHz. The long arm of the interferometer is wound around a fiber-stretcher to remove the effect of low-frequency drifts. The interferometer is closed by another fiber BS, and the output light is detected with a fiber-coupled balanced photodetector (BPD). The BPD's output voltage is sent to the controller, implemented using the I/Q modulator/demodulator module within the RedPitaya/PyRPL FPGA architecture. (b) Phase noise spectra $S_{\dot{\phi}\dot{\phi}}$ for $\gamma_{\rm IQ} = 2\pi \times 4$ kHz and $\Omega_{\rm IQ} = \Omega_\alpha$, measured under different cancellation gains $g$. The inset shows the behavior of phase noise at $\Omega_\alpha$ as a function of $g$, along with the theoretical prediction based on our experimental parameters (black solid line).

## I: CALIBRATION OF QUASI-HOMODYNE DETECTION

This section details the calibration of the detection scheme used to measure the occupations when deploying active phase noise cancellation [Fig. 3(a), squares and triangles]. Detection of the extremely low occupation numbers encountered in our experiments is made possible by a quasi-homodyne detection scheme. To maximize the detection efficiency $\eta$ of our apparatus, the local oscillator frequency of our heterodyne detection is changed from 2 MHz to 9 Hz, creating a quasi-homodyne scheme [15]. This adjustment approximately doubles the detection efficiency while avoiding low frequency drifts that typically require active locking in true homodyne detection. Furthermore, small improvements in the fiber coupling of the light back-scattered by the librator, along with modifications to the DAQ system used to acquire the data and a change in the electronic gain of our detector, increased the information detection efficiency from $\eta \approx 1\%$ (when data without phase noise cancellation was acquired) to $\eta \approx 5\%$ (for data with phase noise cancellation).

We calibrate our quasi-homodyne detection using Raman sideband thermometry with the heterodyne spectrum. During the calibration, we perform sets of measurements in both quasi-homodyne, and heterodyne configurations across different phase noise cancellation gains $g$ to verify the calibration across varying occupation numbers. To mitigate any classical asymmetry from the detector, we swap the heterodyne local oscillator frequency between +2 MHz and −2 MHz. By alternating the sign of the heterodyne local oscillator frequency, we can effectively cancel out contributions from the detector's transfer function [14], which could otherwise distort the measured occupation. In Fig. S10, we show the calibration factors of the quasi-homodyne across all realizations. These calibration factors $C_n$ are determined by taking the ratio between $2n + 1$ (with $n$ determined by a heterodyne measurement) and the area of the Lorentzian fitted on the quasi-homodyne spectra of the photocurrent (in units of A$^2$). To determine the final calibration factor, we compute the average calibration factor across all realizations and combine the uncertainties by taking the mean-squared value of the standard deviations. This yields a final calibration factor of $C_n = 6(2) \times 10^{15}\, n/\mathrm{A}^2$.

## J: HEATING RATE MEASUREMENTS

This appendix presents independent measurements to extract the libration heating rate $\Gamma$. To directly measure $\Gamma$ starting from the ground state, we abruptly turn the phase noise eater off and follow the occupation of the oscillator as a function of time. During this switching, we monitor $i_\alpha$, the current output of our detector, demodulated at $\Omega_\alpha$. In Fig. S11(a), we show a time trace of $i_\alpha$, as we switch the gain of the phase noise eater from $g = 0.4$ (gray areas) to $g = 0$ (white areas).

To determine the occupation number $n$ from $i_\alpha$, we first calculate $i_\alpha^2 - i_{\rm sn}^2$, where $i_{\rm sn}$ is the shot-noise contribution to the demodulated current. The shot-noise current $i_{\rm sn}$ is measured independently by demodulating the detector signal while blocking the light scattered by the particle. We then calibrate $i_\alpha^2 - i_{\rm sn}^2$ with our quasi-homodyne detection. In Fig. S11(b), we show the extracted

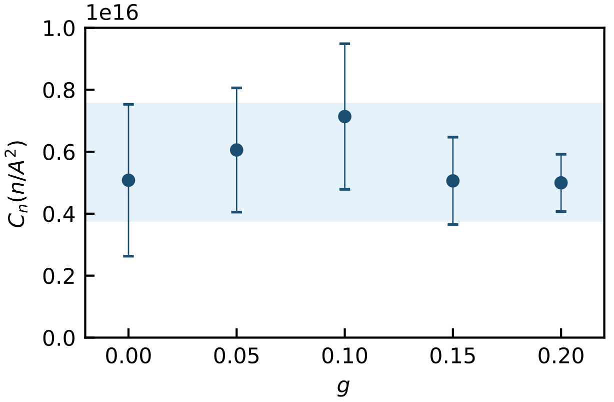


FIG. S10. **Quasi-homodyne calibration factor.** Calibration factor $C_n$ of the quasi-homodyne signal as a function of phase noise suppression factor $g$. The values of $C_n$ are obtained by computing the ratio of the quantity $2n + 1$, where $n$ is obtained from sideband asymmetry, to the area of the homodyne Lorentzian peak, in Ampere squared. Errorbars are obtained by propagating the errors from the estimations of $n$ and the area of the homodyne peak. Shaded area is the average calibration factor across all five different realizations, and the thickness of the area represents one standard deviation from the mean.

average occupation number $\bar{n}$ changing over time $t$, with $t = 0$ corresponding to the instant of switching to $g = 0$. The data is averaged over 500 iterations of the switching protocol. With the phase noise eater engaged ($g = 0.4$), the libration thermalizes to the ground state ($n < 1$, gray area). After switching to $g = 0$ at $t = 0$, the averaged occupation number $\bar{n}$ increases. From a linear fit (dashed black line) we extract a total heating rate of $\dot{\bar{n}} = 133(1) \times 10^3\ \mathrm{s}^{-1}$, in good agreement with the steady-state heating rate estimation $\Gamma = 121(4) \times 10^3\ \mathrm{s}^{-1}$ obtained from $\Gamma = \gamma \times n$.

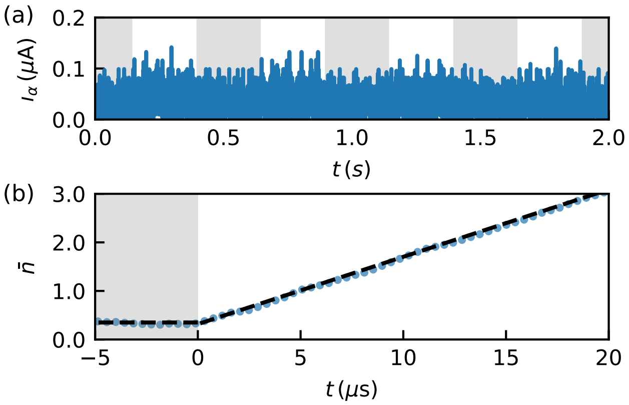


FIG. S11. **Direct measurement of phase noise heating rate.** (a) Demodulated current of the homodyne detector $i_\alpha$ as we switch the gain of the phase noise eater from $g = 0.4$ (gray areas) to $g = 0$ (white areas) over time $t$. (b) Average occupation number $\bar{n}$ extracted from 500 repetitions of the switch protocol (blue dots). The phase noise eater is turned off ($g = 0$) at time $t = 0$, revealing a constant heating rate of $\dot{\bar{n}} = 133(1) \times 10^3\ \mathrm{s}^{-1}$ (black dashed line).


[1] M. Kamba, R. Shimizu, and K. Aikawa, Nanoscale feedback control of six degrees of freedom of a near-sphere, Nat. Comm. **14**, 7943 (2023).

[2] C. Gonzalez-Ballestero, J. Zielińska, M. Rossi, A. Militaru, M. Frimmer, L. Novotny, P. Maurer, and O. Romero-Isart, Suppressing recoil heating in levitated optomechanics using squeezed light, PRX Quantum **4**, 030331 (2023).

[3] C. Gonzalez-Ballestero, P. Maurer, D. Windey, L. Novotny, R. Reimann, and O. Romero-Isart, Theory for cavity cooling of levitated nanoparticles via coherent scattering: Master equation approach, Phys. Rev. A **100**, 013805 (2019).

[4] C. Gonzalez-Ballestero, Tutorial: projector approach to master equations for open quantum systems, Quantum **8**, 1454 (2024).

[5] I. Wilson-Rae, N. Nooshi, J. Dobrindt, T. J. Kippenberg, and W. Zwerger, Cavity-assisted backaction cooling of mechanical resonators, New Journal of Physics **10**, 095007 (2008).
[6] N. Van Kampen, A cumulant expansion for stochastic linear differential equations. i, Physica **74**, 215 (1974).
[7] N. Van Kampen, A cumulant expansion for stochastic linear differential equations. ii, Physica **74**, 239 (1974).
[8] F. van der Laan, F. Tebbenjohanns, R. Reimann, J. Vijayan, L. Novotny, and M. Frimmer, Sub-kelvin feedback cooling and heating dynamics of an optically levitated librator, Phys. Rev. Lett. **127**, 123605 (2021).
[9] P. Rabl, C. Genes, K. Hammerer, and M. Aspelmeyer, Phase-noise induced limitations on cooling and coherent evolution in optomechanical systems, Phys. Rev. A **80**, 063819 (2009).
[10] U. Delić, M. Reisenbauer, D. Grass, N. Kiesel, V. Vuletić, and M. Aspelmeyer, Cavity cooling of a levitated nanosphere by coherent scattering, Phys. Rev. Lett. **122**, 123602 (2019).
[11] N. Meyer, A. d. l. R. Sommer, P. Mestres, J. Gieseler, V. Jain, L. Novotny, and R. Quidant, Resolved-sideband cooling of a levitated nanoparticle in the presence of laser phase noise, Phys. Rev. Lett. **123**, 153601 (2019).
[12] J. Piotrowski, D. Windey, J. Vijayan, C. Gonzalez-Ballestero, A. de los Ríos Sommer, N. Meyer, R. Quidant, O. Romero-Isart, R. Reimann, and L. Novotny, Simultaneous ground-state cooling of two mechanical modes of a levitated nanoparticle, Nat. Phys. **19**, 1009 (2023).
[13] D. Windey, C. Gonzalez-Ballestero, P. Maurer, L. Novotny, O. Romero-Isart, and R. Reimann, Cavity-based 3d cooling of a levitated nanoparticle via coherent scattering, Phys. Rev. Lett. **122**, 123601 (2019).
[14] F. Tebbenjohanns, M. Frimmer, V. Jain, D. Windey, and L. Novotny, Motional sideband asymmetry of a nanoparticle optically levitated in free space, Phys. Rev. Lett. **124**, 013603 (2020).
[15] J. Gao, F. van der Laan, J. A. Zielińska, A. Militaru, L. Novotny, and M. Frimmer, Feedback cooling a levitated nanoparticle's libration to below 100 phonons, Phys. Rev. Res. **6**, 033009 (2024).
[16] F. van der Laan, R. Reimann, A. Militaru, F. Tebbenjohanns, D. Windey, M. Frimmer, and L. Novotny, Optically levitated rotor at its thermal limit of frequency stability, Phys. Rev. A **102**, 013505 (2020).
[17] M. Parniak, I. Galinskiy, T. Zwettler, and E. S. Polzik, High-frequency broadband laser phase noise cancellation using a delay line, Opt. Express **29**, 6935 (2021).
